# The Effect of Oxidative Stress and Memantine-Incorporated Reactive Oxygen Species-Sensitive Nanoparticles on the Expression of *N*-Methyl-d-aspartate Receptor Subunit 1 in Brain Cancer Cells for Alzheimer’s Disease Application

**DOI:** 10.3390/ijms222212309

**Published:** 2021-11-15

**Authors:** Jung Sun Park, Taeyeon Kim, Dohoon Kim, Young-IL Jeong

**Affiliations:** 1 Department of Internal Medicine, Chonnam National University Medical School, 42 Jebongro, Gwangju 61469, Korea; gene-pjs@hanmail.net; 2 College of Art&Science, University of Pennsylvania, 249 S 36th St., Philadelphia, PA 19104, USA; taeyeonk@sas.upenn.edu; 3 Department of Integrative Physiology and Pathobiology, Tufts University School of Medicine, Boston, MA 02111, USA; DoHoon.Kim@tufts.edu; 4 Research Institute of Convergence of Biomedical Sciences, Pusan National University Yangsan Hospital, Yangsan 50612, Korea

**Keywords:** Alzheimer’s disease, reactive oxygen species, memantine, cyclodextrin nanoparticles, ROS-sensitive drug delivery

## Abstract

The aim of this study is to fabricate reactive oxygen species (ROS)-sensitive nanoparticles composed of succinyl β-cyclodextrin (bCDsu), memantine and thioketal linkages for application in Alzheimer’s disease, and to investigate the suppression of *N*-methyl-d-aspartate (NMDA) receptor 1 (NMDAR1) in cells. Thioketal diamine was attached to the carboxyl group of bCDsu to produce thioketal-decorated bCDsu conjugates (bCDsu-thioketal conjugates) and memantine was conjugated with thioketal dicarboxylic acid (memantine-thioketal carboxylic acid conjugates). Memantine-thioketal carboxylic acid conjugates were attached to bCDsu-thioketal conjugates to produce bCDsu-thioketal-memantine (bCDsuMema) conjugates. SH-SY5Y neuroblastoma cells and U87MG cells were used for NMDAR1 protein expression and cellular oxidative stress. Nanoparticles of bCDsuMema conjugates were prepared by means of a dialysis procedure. Nanoparticles of bCDsuMema conjugates had small particle sizes less than 100 nm and their morphology was found to be spherical in transmission electron microscopy observations (TEM). Nanoparticles of bCDsuMema conjugates responded to H_2_O_2_ and disintegrated or swelled in aqueous solution. Then, the nanoparticles rapidly released memantine according to the concentration of H_2_O_2_. In an in vivo animal imaging study, thioketal-decorated nanoparticles labelled with fluorescent dye such as chlorin e6 (Ce6) showed that the fluorescence intensity was stronger in the brain than in other organs, indicating that bCDsuMema nanoparticles can efficiently target the brain. When cells were exposed to H_2_O_2_, the viability of cells was time-dependently decreased. Memantine or bCDsuMema nanoparticles did not practically affect the viability of the cells. Furthermore, a western blot assay showed that the oxidative stress produced in cells using H_2_O_2_ increased the expression of NMDAR1 protein in both SH-SY5Y and U87MG cells. Memantine or bCDsuMema nanoparticles efficiently suppressed the NMDAR1 protein, which is deeply associated with Alzheimer’s disease. Fluorescence microscopy also showed that H_2_O_2_ treatment induced green fluorescence intensity, which represents intracellular ROS levels. Furthermore, H_2_O_2_ treatment increased the red fluorescence intensity, which represents the NMDAR1 protein, i.e., oxidative stress increases the expression of NMDAR1 protein level in both SH-SY5Y and U87MG cells. When memantine or bCDsuMema nanoparticles were treated in cells, the oxidative stress-mediated expression of NMDAR1 protein in cells was significantly decreased, indicating that bCDsuMema nanoparticles have the capacity to suppress NMDAR1 expression in brain cells, which has relevance in terms of applications in Alzheimer’s disease.

## 1. Introduction

Oxidative stress in the human body is associated with inflammatory pathways and the progression of various kinds of disease such as cancer, neurodegenerative disease, diabetes and hypertension [1,2,3,4,5,6]. Among them, oxidative stress has a diverse relationship with the severity and progression of neurodegenerative disease such as Alzheimer’s disease, Parkinson’s disease, many other neural disorders and aging [4,7,8]. An imbalance of reactive oxygen species (ROS) induces the degeneration of biomolecules such as DNA, proteins and lipids, which leads to the neurodegeneration and/or apoptotic death of neuronal cells [7,8,9]. Paradoxically, oxidative stress derived from elevated levels of ROS can be considered as a biomarker since various molecular receptors including the *N*-methyl-d-aspartate (NMDA) receptor can be altered through the molecular pathway of oxidative stress [10,11,12,13,14]. For example, the NMDA receptors in neuronal and/or brain endothelial cells are known to be upregulated according to the increase in oxidative stress [13,15]. Betzen et al. reported that oxidative stress in the cerebrovascular endothelium is associated with disruption of the blood–brain barrier (BBB) [13]. The presence of NMDA receptor upregulation induced by oxidative stress can be used as a biomarker for Alzheimer’s disease [15]. Furthermore, oxidative stress can be used to diagnose and target Alzheimer’s disease [16]. Otherwise, various kinds of agents have been developed to treat Alzheimer’s disease [17,18,19,20,21]. Among them, NMDA inhibitors have been investigated as one of the solutions for the treatment of Alzheimer’s disease since the activation of NMDA receptors and amyloid-β (Aβ) toxicity is associated with synapse density and memory formation [22]. There is no curative option for Alzheimer’s disease up to now and these regimens are still limited to palliative therapy. Regarding these regimens, unwanted side effects of therapeutic agents for Alzheimer’s disease including memantine, a typical NMDA antagonist, are frequently problematic in clinical trials [23,24]. Furthermore, the blood–brain barrier (BBB) is still considered as an obstacle for the brain delivery of therapeutic agents even though BBB function is normally disrupted in patients of Alzheimer’s disease [25,26]. These hurdles make difficult to manage Alzheimer’s disease.

Nano-dimensional carriers such as nanoparticles, liposomes, peptide-drugs and/or polymeric drugs have been investigated for the improvement of drug delivery across the BBB [27,28,29,30]. Nano-dimensional carriers are characterized by small particle sizes, diversity of customized form, ease of lipophilic drug encapsulation, increased half lives in the body, and targeting of the drug to specific sites of action [31]. In particular, nanoparticles are considered as a promising device to treat neuro-degenerative disease through enhanced drug delivery across the BBB [32,33,34,35]. Liu et al. reported that zeolitic imidazolate framework 8-coated Prussian blue nanocomposite could penetrate the BBB and then released quercetin [34]. They showed that nanocomposites significantly increased the level of adenosine phosphate, reduced the oxidative stress and reversed dopaminergic neuronal damage. Berberine-encapsulated mesoporous silica nanoparticles effectively inhibited amyloid fibrillation and decreased the level of malondialdehyde [36]. Lee et al. reported that redox-responsive nanoparticles have sensitivity to ROS formation in cancer cells and that the release rate of anticancer drugs can be controlled by intracellular oxidative stress in cancer cells [37]. Stimuli-sensitive nanoparticles, which sensitively respond to the microenvironments of specific organ/tissues and then release the bioactive agents, have great potential for drug targeting because they can specifically deliver cytotoxic agents to disease sites while minimizing unwanted side-effects against normal tissues or organs [37,38,39,40]. For example, antibacterial agents could be specifically released from nanoparticles with ROS-sensitive linkages when the ROS levels were increased in the urinary tract by bacterial infection [38]. It was found that the ligand-peptide of low-density lipoprotein receptor-decorated nanoparticles can penetrate into brain across the BBB and then effectively reduce the activation of microglia cells [39]. Balance et al. showed that particle-based drug delivery systems can be designed in response to ROS levels in the disease sites of neurological disorders [40].

In this study, we designed ROS-sensitive nanoparticles for the brain delivery of NMDA antagonists. For this purpose, we synthesized succinyl β-cyclodextrin-thioketal-memantine conjugates for the fabrication of the ROS-specific delivery of memantine followed by the inhibition of NMDA receptors. Since the thioketal linkage can be disintegrated in the presence of ROS, the thioketal linkage was introduced in a conjugated form to liberate memantine in an ROS-specific manner [40]. We investigated the efficacy of nanoparticles in terms of brain delivery and NMDA inhibition.

## 2. Results

### 2.1. Synthesis of bCDsuMema Conjugates

**Figure 1:**
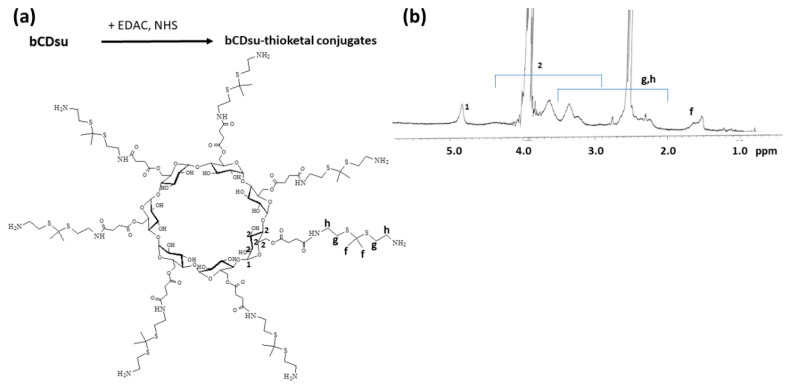
(**a**) Synthesis scheme and (**b**) ^1^H NMR spectra of bCD-thioketal amine. To measure chemical structure with ^1^H NMR spectroscopy, bCDsu-thioketal amine was dissolved in dimethyl sulfoxide (DMSO)-d form. bCDsu, the ^1^H NMR spectra of bCDsu and thioketal diamine are shown in Figure S1.

**Figure 2:**
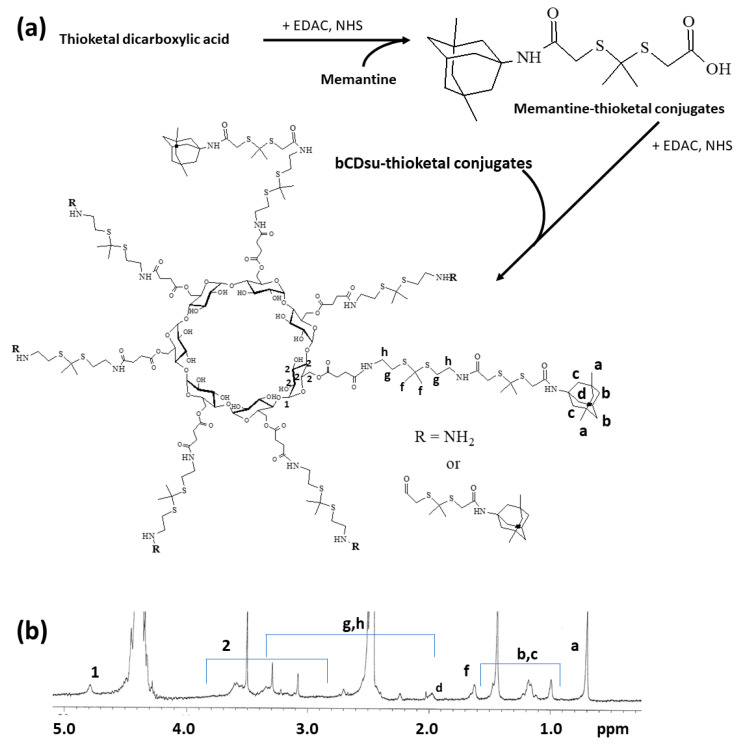
(**a**) Synthesis scheme and (**b**) ^1^H NMR spectra of bCDsuMema conjugates. The ^1^H NMR spectra of memantine and thioketal dicarboxylic acid are shown in Figure S1.

To synthesize bCDsuMema conjugates, bCDsu was conjugated with thioketal diamine, as shown in Figure 1a. The carboxylic acid group of bCDsu was activated with the EDAC/NHS system and then an excess amount of thioketal diamine was added. Specific peaks of bCDsu were confirmed between approximately 2 and 5 ppm, while specific peaks of thioketal diamine, such as those of the amine groups, were confirmed at 1.6 ppm for the CH_3_ group and 8.4 ppm for the CH_2_ group, respectively. Furthermore, specific peaks of thioketal dicarboxylic acid were also confirmed at 1.6 ppm and 12.6 ppm, respectively, as shown in Figure S1. Synthesis of bCDsu-thioketal conjugates was confirmed using ^1^H NMR spectra, as shown in Figure 1b. The specific peaks of bCDsu-thioketal were obtained between approximately 1.0 and 5.0 ppm, i.e., the methyl proton of the thioketal group was observed at 1.4~1.6 ppm, while specific peaks of bCDsu were confirmed between approximately 2.0 and 5.0 ppm, as shown in Figure 1b. Prior to attaching memantine to these conjugates, memantine was primarily conjugated with thioketal dicarboxylic acid to produce memantine-thioketal carboxylic acid conjugates, as shown in Figure 2a. The carboxylic group of the memantine-thioketal carboxylic acid conjugates was activated with the EDAC/NHS system, and was then conjugated with bCDsu-thioketal conjugates, as shown in Figure 2a. As shown in Figure 2b, specific peaks of bCDsu, the thioketal group and memantine were confirmed between approximately 0 and 5.0 ppm. Peaks of bCDsu were observed between approximately 2.5 and 5.0 ppm, while specific peaks of the thioketal group and memantine were obtained at 1.6/1.8~3.4 ppm and 1.7~1.8 ppm/0.9~1.5 ppm, respectively (Figure 1b). As shown in Figure S1, the proton NMR spectra of memantine itself was confirmed at 0.8~8.4 ppm. These results showed that the bCDsuMema conjugates were successively synthesized.

### 2.2. Fabrication and Characterization of Nanoparticles

**Figure 3:**
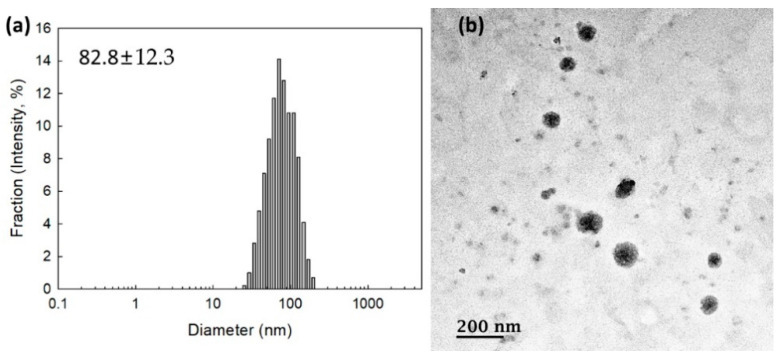
(**a**) Typical particle size distribution and (**b**) TEM photo of bCDsuMema nanoparticles.

To measure the contents of memantine, nanoparticles were incubated with excess amounts of H_2_O_2_ and then dissolved in DMSO. As shown in Table 1, the experimental contents of memantine in the bCDsuMema conjugates were slightly lower than the theoretical levels. To check whether or not the bCDsuMema conjugates formed nano-sized vehicles, bCDsuMema conjugates were dissolved in DMSO/water mixtures and then dialyzed against water. Since they formed a slightly turbid aqueous solution, they were used for the characterization of nanoparticles of bCDsuMema conjugates as well as in a drug release study. As shown in Figure 3a, the diameters of the bCDsuMema conjugates and the average particle sizes were less than 100 nm, i.e., the bCDsuMema nanoparticles showed monomodal size distributions and their average particle sizes were 82.8 ± 12.3 nm. Furthermore, when their morphologies were observed with TEM, they showed almost spherical shapes, as shown in Figure 3b. These results indicated that bCDsuMema conjugates formed spherical nanoparticles in the aqueous solution.

**Figure 4:**
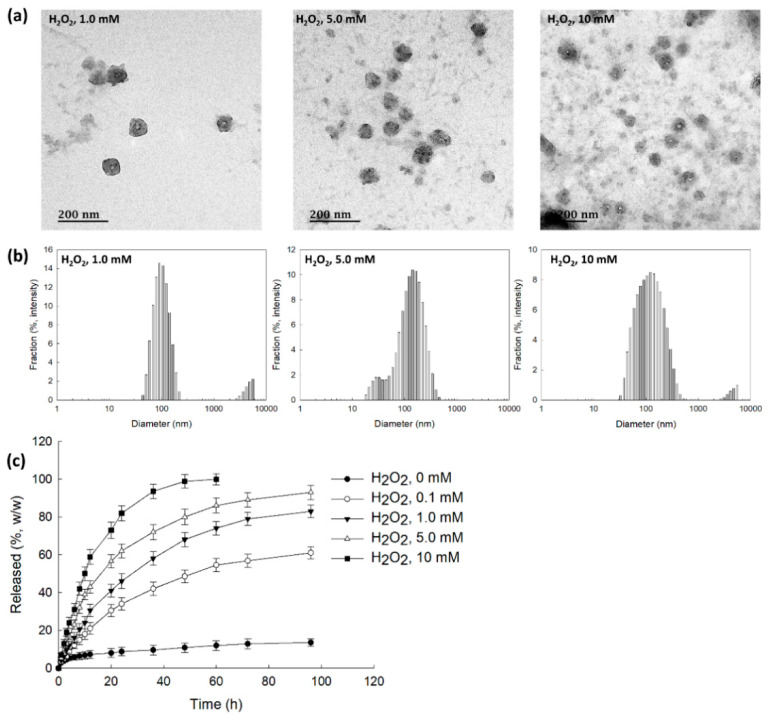
(**a**) The effect of H_2_O_2_ on the morphological changes of bCDsuMema nanoparticles. (**b**) The effect of H_2_O_2_ on the particle size distribution of bCDsuMema nanoparticles. (**c**) The effect of H_2_O_2_ on the release of memantine from nanoparticles. The memantine concentration was adjusted to 0.1 mg/mL in PBS with or without H_2_O_2_.

To assess ROS sensitivity, bCDsuMema nanoparticles were incubated with PBS solution in the presence of H_2_O_2_, as shown in Figure 4. As can be seen in Figure 4a, according to the H_2_O_2_ concentrations, the boundaries of the nanoparticles developed indistinct shapes in the presence of H_2_O_2_, while their boundaries otherwise showed relatively distinct shapes. Furthermore, in the presence of H_2_O_2_, debris of nanoparticles was observed, and this increased with the increasing of the H_2_O_2_ contents. These results indicated that the nanoparticles could be disintegrated by oxidative stress. When H_2_O_2_ was added, the size distribution became relatively broad and a multi-modal pattern appeared as compared to the distribution observed in the absence of H_2_O_2_, as shown in Figure 4b. These results indicated that bCDsuMema nanoparticles responded to the ROS environment in the aqueous solution. The addition of H_2_O_2_ induced the acceleration of the release of memantine from bCDsuMema nanoparticles, as shown in Figure 4c. In the absence of H_2_O_2_, the liberation of memantine from nanoparticles was less than 20% (*w/w*) for 96 h, while more than 80% (*w/w*) and 90% (*w/w*) of memantine in the nanoparticles was released at H_2_O_2_ concentrations higher than 5 mM and 10 mM, respectively. These results indicated that bCDsuMema nanoparticles have ROS-sensitivity and ROS-sensitive drug release capacity in aqueous environments. The properties of size distribution shown in Figure 4b are abbreviated in Table 2. As shown in Table 2, the main peaks of the particle sizes were relatively increased according to the increase in H_2_O_2_ contents, and second peaks were also observed.

### 2.3. In Vivo Biodistribution of Nanoparticles

**Figure 5:**
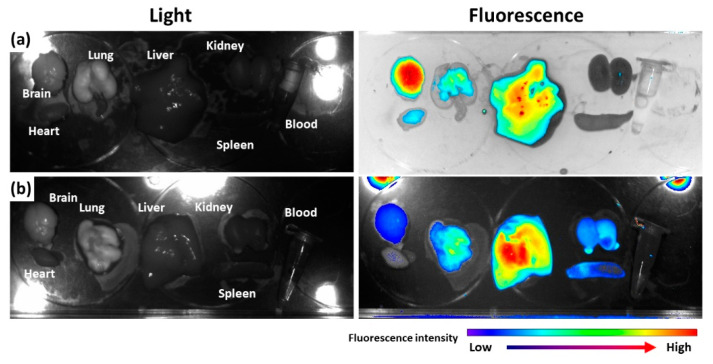
Biodistribution of bCDsuTHCe6 nanoparticles (**a**) and bCDsuCe6 nanoparticles (**b**). The synthesis schemes of bCDsuTHCe6 and bCDsuCe6 nanoparticles are illustrated in Figure S2. For fluorescence imaging of nanoparticles, nude BALb/C mice (male, 20 g, 5 weeks old) were used. bCDsuTHCe6 or bCDsuCe6 nanoparticles were intravenously administered via the tail vein of the mice. The injection dose was 10 mg Ce6/kg mouse. Injection solution was sterilized with a 0.8 µm syringe filter. Injection volume was 100 µL. After 24 h of injection, the mice were sacrificed and then each organ was observed with a Maestro^TM^ 2 small animal imaging instrument.

To assess the in vivo fate of nanoparticles, Ce6, as a near-infrared fluorescent dye, was attached to bCDsu-thioketal conjugates (bCDsuTHCe6), as shown in Figure S2a. Furthermore, Ce6-thioketal amine was prepared and conjugated with bCDsu (bCDsuCe6), as shown in Figure S2b. Figure 5 shows the biodistribution of bCDsuTHCe6 nanoparticles (Figure 5a) and bCDsuCe6 nanoparticles (Figure 5b). As shown in Figure 5a, the fluorescence intensity of bCDsuTHCe6 nanoparticles was significantly higher in the brain than that in other organs. Otherwise, bCDsuCe6 nanoparticles revealed lower fluorescent intensity in the brain compared to bCDsuTHCe6 nanoparticles. In the case of bCDsuTHCe6 nanoparticles, the fluorescence intensity was stronger in the brain and liver while the fluorescence intensity of the bCDsuCe6 nanoparticles was relatively lower in the brain than that in other organs. These results indicated that bCDsu-thioketal conjugates have excellent brain drug delivery.

### 2.4. The Effect of Oxidative Stress on the NMDAR1 Expression in SH-SY5Y Neuroblastoma Cells and U87MG Cells

**Figure 6:**
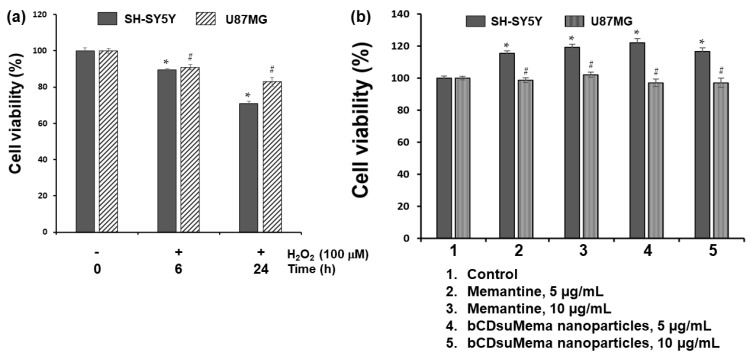
(**a**) The effect of H_2_O_2_ on the viability of cells. (**b**) The effect of memantine and bCDsuMema nanoparticles on the viability of cells. Cell (1 × 10^4^ cells in 96 wells) were exposed to H_2_O_2_ (final concentration: 100 µM) in serum-free media for 6 h or 24 h. For treatment of memantine or nanoparticles, cells were treated with 5 and 10 µg/mL of memantine or bCDsuMema nanoparticles for 24 h. All cell culture experiments were triplicated and then expressed as average ± standard deviation (S.D.). * *p* < 0.05, compared with the SH-SY5Y cell control. # *p* < 0.05, compared with the U87MG cell control.

**Figure 7:**
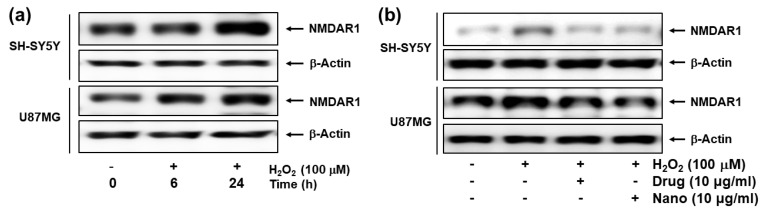
(**a**) The effect of H_2_O_2_ on the NMDAR1 expression of cells. (**b**) The effect of memantine and bCDsuMema nanoparticles on the NMDAR1 expression of cells. Cells were pre-treated with memantine, or nanoparticles were treated for 1 h and then exposed to H_2_O_2_ (final concentration: 100 µM) for 6 h. Drug = memantine; Nano = bCDsuMema nanoparticles.

**Figure 8:**
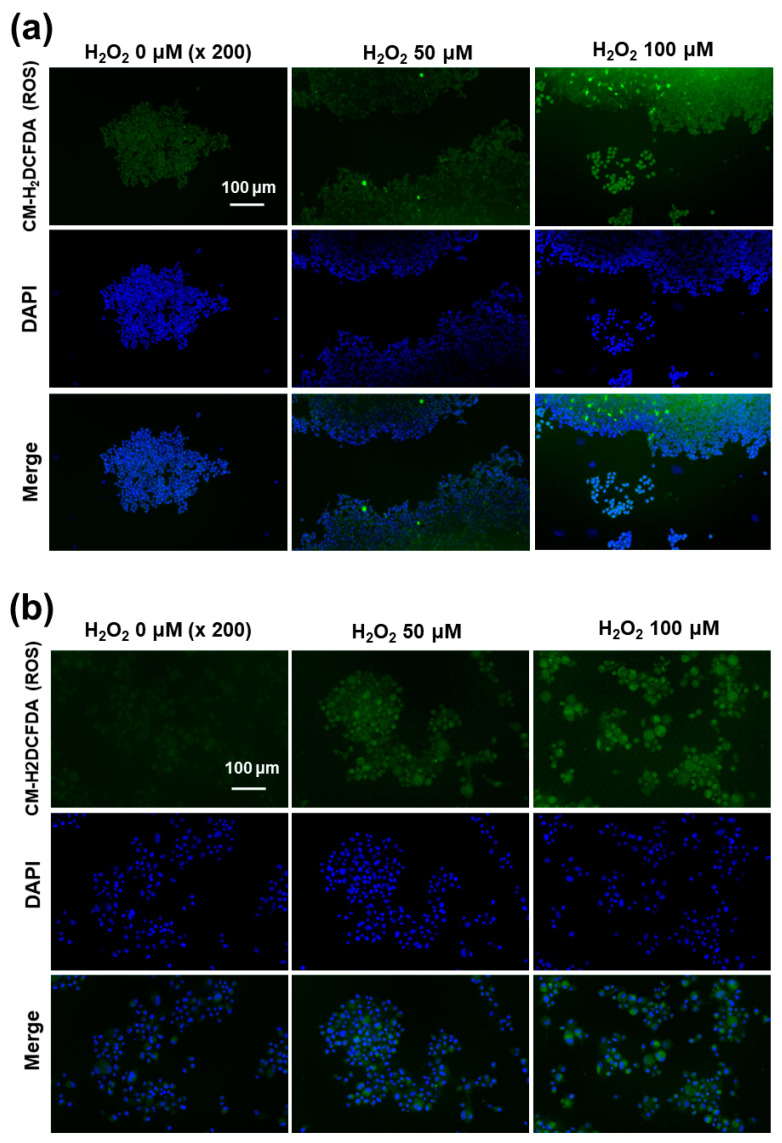
Fluorescence observation of SH-SY5Y cells (**a**) and U87MG cells (**b**). The effect of H_2_O_2_ on the intracellular ROS intensity. U87MG cells (1 × 10^5^) or SH-SY5Y cells (1 × 10^5^) were treated with 100 μM H_2_O_2_ for 6 h. After that, cells were stained with CM-H_2_DCFDA.

**Figure 9:**
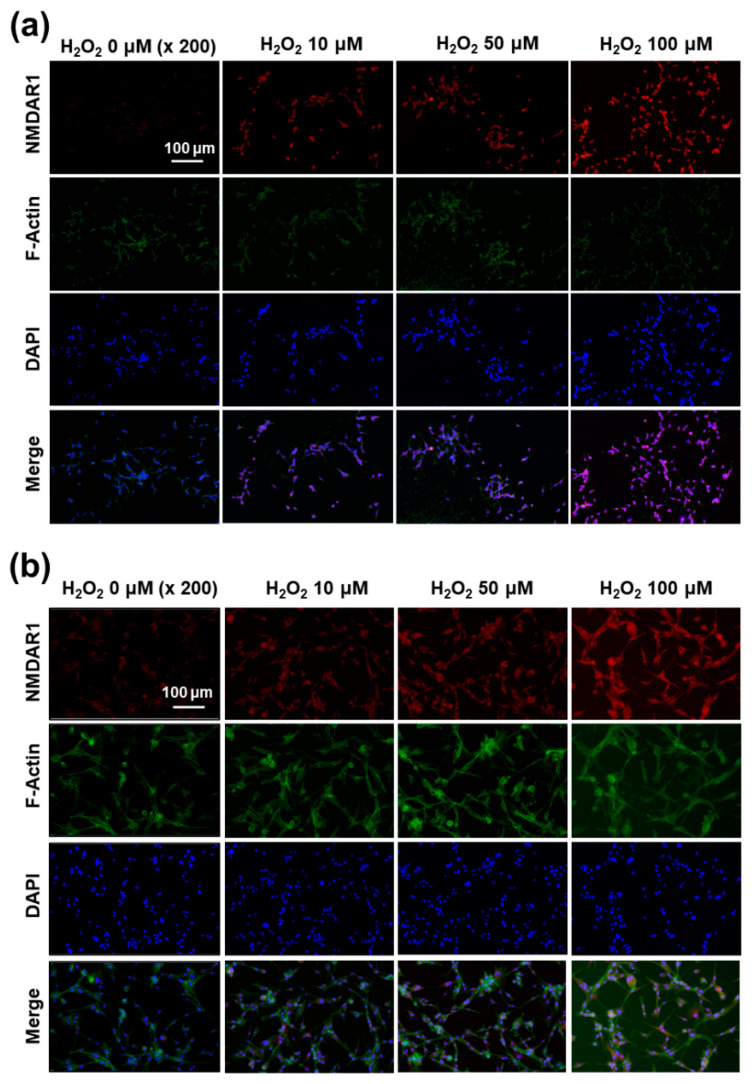
The effect of oxidative stress on the expression of NMDAR1 at SH-SY5Y cells (**a**) and U87MG cells (**b**). SH-SY5Y cells (1 × 10^5^) or U87MG cells (1 × 10^5^) were treated with H_2_O_2_ for 6 h. After that, cells were fixed with 4% paraformaldehyde and stained with anti-NMDAR1 antibody. The cells were incubated with a Cy3 (red fluorescence) goat anti-rabbit antibody diluted 1:500 in blocking buffer for 2 h, and a Phalloidin was added for incubation (F-actin, Alexa Fluor 488, green fluorescence) for 30 min. The use of red, green and blue color represents the staining of the NMDAR1 protein, actin and nucleus in cells.

**Figure 10:**
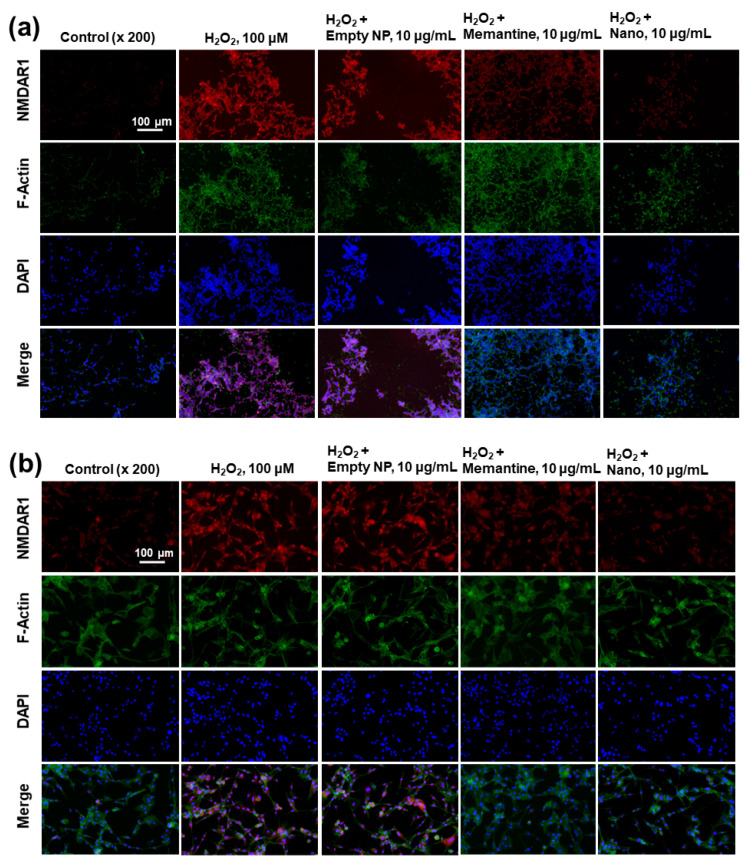
The effect of memantine and/or bCDsuMema nanoparticles on the expression of NMDAR1 in SH-SY5Y cells (**a**) and U87MG cells (**b**) under oxidative stress. For the observation of NMDAR1 changes of cells, U87MG cells (1 × 10^5^) or SH-SY5Y cells (1 × 10^5^) were pre-treated with memantine or bCDsuMema nanoparticles for 1 h and then treated with 100 μM H_2_O_2_ for 6 h. Immunofluorescence staining of cells was performed in a similar manner to that shown in Figure 9. Nano = bCDsuMema nanoparticles.

To study the effect of ROS on the viability and NMDAR1 protein expression, H_2_O_2_ was exposed to SH-SY5Y cells and U87MG cells as shown in Figure 6, Figure 7, Figure 8, Figure 9 and Figure 10. Figure 6 shows the effect of H_2_O_2_, memantine and bCDsuMema nanoparticles on the viability of SH-SY5Y cells and U87MG cells. As shown in Figure 6a, human neuroblastoma SH-SY5Y and glioma U87MG cells treated with 100 μM H_2_O_2_ for 0 h, 6 h, or 24 h resulted in a time-dependent decrease in viability. On the other hand, memantine or bCDsuMema nanoparticles with 5 or 10 μg/mL memantine concentration had no cytotoxicity against SH-SY5Y and U87MG cells (Figure 6b). These findings suggested that the viability of SH-SY5Y and U87MG cells was affected by ROS, but not by memantine and bCDsuMema nanoparticles.

Figure 7 shows the effect of H_2_O_2_ (100 μM) treatment on the expression of the NMDAR1 protein in SH-SY5Y and U87MG cells. As shown in Figure 7a, treatment with H_2_O_2_ induced in increase in NMDAR1 protein expression in both SH-SY5Y and U87MG cells in a time-dependent manner. When memantine or bCDsuMema nanoparticles were treated, the expression levels of NMDAR1 proteins in both SH-SY5Y and U87MG cells were decreased as compared to H_2_O_2_ treatment only (Figure 7b). These results indicated that NMDAR1 protein expression was time-dependently increased when cells were exposed to ROS. However, the increase in the cellular expression of NMDAR1 protein can be efficiently reversed by treatment with memantine. Furthermore, these results showed that, as well as memantine, the bCDsuMema nanoparticles also possessed the capability to suppress the NMDAR1 protein in cells.

Since the western blot assay showed ROS-mediated expression of the NMDAR1 protein, the intracellular ROS formations in the SH-SY5Y and U87MG cells were stained using the ROS-sensitive fluorescent dye CM-H_2_DCFDA and then observed with fluorescence microscopy, as shown in Figure 8. As shown in Figure 8a, the green fluorescence intensity was gradually increased according to the concentration of H_2_O_2_, indicating that the intracellular level of ROS gradually increased with incubation of cells using H_2_O_2_. Furthermore, the intracellular ROS levels in U87MG cells were also increased according to the concentration of H_2_O_2_ (Figure 8b). These results indicated that the intracellular ROS levels were increased by pretreatment with H_2_O_2_ and then affected by the physiological changes of the cellular component.

Since H_2_O_2_ pretreatment induces the expression of the NMDAR1 protein in SH-SY5Y and U87MG cells, as shown in Figure 7a, the expression of the NMDAR1 protein in cells was fluorescently observed, as shown in Figure 9. As shown in Figure 9a, the pretreatment of H_2_O_2_ induced the increase in red fluorescence intensity in SH-SY5Y cells, i.e., the expression level of the NMDAR1 protein was dose-dependently increased according to the increase in H_2_O_2_ concentration. In addition, NMDAR1 protein expression in U87MG cells were also increased dose-dependently according to the concentration of H_2_O_2_, as shown in Figure 9b. These results indicated that H_2_O_2_ treatment clearly induces the expression of NMDAR1 protein. These results support the results shown in Figure 7a and, furthermore, indicated that oxidative stress induces the expression of the NMDAR1 protein in neuronal cells.

The effect of memantine and/or bCDsuMema nanoparticles on the expression of the NMDAR1 protein in cells was observed fluorescently, as shown in Figure 10. Since memantine or bCDsuMema nanoparticles efficiently suppressed the ROS-mediated expression of NMDAR1 protein in SH-SY5Y or U87MG cells, as shown in Figure 7b, the cells were stained fluorescently and the changes in their NMDAR1 expression were observed with a fluorescence microscope, as shown in Figure 10. As shown Figure 10a, the red fluorescence intensity efficiently decreased as a result of treatment with memantine or bCDsuMema nanoparticles, i.e., the H_2_O_2_-induced expression of NMDAR1 in SH-SY5Y cells gradually decreased following treatment of Mema or bCDsuMema nanoparticles. These results also support the findings shown in Figure 7b, and indicated that bCDsuMema nanoparticles efficiently suppress the ROS-mediated expression of the NMDAR1 protein in cells. As shown in Figure 10b, the ROS-induced NMDAR1 protein expression in U87MG cells was also suppressed by treatment with memantine or bCDsuMema nanoparticles. These results indicated that bCDsuMema nanoparticles are able to suppress the oxidative-stress-induced expression of the NMDAR1 protein in SH-SY5Y neuroblastoma cells or U87MG glioblastoma cells as well as free memantine.

## 3. Discussion

The physiological state of the brain is quite different to that of other organs because the BBB is a primary obstacle for drug delivery to the brain [25,26]. For this reason, various delivery platforms have been investigated to improve the penetration and/or transport of bioactive agents [26,27,28,29,30,31,32,33,34,35,36,41,42,43,44,45,46,47]. For example, surfactants such as polysorbate 80 have been used to allow nanoparticles or drug carriers to penetrate the BBB [41,42,43,44,45,46]. For example, Alyaudtin et al. reported that poly(butylcyanoacrylate) nanoparticles overcoated with polysorbate 80 can be used for interaction with the BBB and can be used for brain drug delivery [41]. They found that polysorbate 80-coated nanoparticles can be efficiently delivered to rat cerebral endothelial cells, but un-coated nanoparticles cannot. Koffie et al. reported that polysorbate 80-coated nanoparticles were used to deliver BBB-impermeable molecules with various molecular weights from 500-Da to 150,000-Da tagged immunoglobulins into the mouse brain [43]. Monoclonal antibodies such as OX-26 can be used to decorate nanoparticles for the brain delivery of bioactive agents [44]. Liu et al. reported that zeolite imidazolate framework 8-based nanocomposites delivered quercetin to the brain, reduced oxidative stress and reversed dopaminergic neuronal damage [34]. They argued that nanocomposites can be used for neurodegenerative diseases without any damage to the normal tissues according to the results found in a mouse model. Amino acid-based molecules were also studied to in relation to BBB transport and to delivery of anticancer agents [45,46]. Furthermore, poly-amine materials such as polyamidoamine (PAMAM) dendrimers were also reported as a platform for brain drug delivery [47,48,49]. Fana et al. reported that PAMAM nanocarriers can be used to deliver bioactive molecules for the treatment of glioblastoma [48]. Pereira et al. reported that recombinant-precursor microRNA (pre-miR-29b) was delivered to the brain with the aid of nanocarriers based on chitosan/polyethyleneimine (PEI) [49]. They argued that chitosan-based nanocarriers can deliver pre-miR-29b across the BBB more efficiently than PEI-based carriers. Positively charged cationic polymers could promote electrostatic interactions with negatively charged RBE cells and then transport bioactive agents across the BBB. In our study, the bCDsuTHCe6, amine-group-decorated conjugates could be efficiently delivered to the brain, and then showed significantly higher fluorescence intensity, while the bCDsuCe6 conjugates had higher fluorescence intensity in other organs, as shown in Figure 5. These results indicated that thioketal amine-decorated bCDsuMema nanoparticles can also be delivered to the brain for applications in Alzheimer’s disease. Sánchez-López et al. reported that memantine-loaded PEGylated polylactic-co-glycolic (PLGA) nanoparticles showed a slower release profile and then showed a reduced drug-administration frequency [50]. They argued that memantine-loaded PLGA/PEG nanoparticles were delivered to the brain across the BBB and, in behavioral tests using transgenic APPswe/PS1dE9 mice, were demonstrated to enhance the benefit of decreased memory impairment compared to the free memantine. In addition, their memantine-loaded nanoparticles suitably reduced the β-amyloid plaques and the associated inflammation caused by Alzheimer’s disease.

Imbalances in ROS levels in the biological system induce oxidative stress and then induce various neurodegenerative disorders [9]. Oxidative stress is known to have a strong relationship with the progression of Alzheimer’s disease and, furthermore, upregulates the expression of NMDA receptors on the cerebrovascular endothelium, which is a biomarker of Alzheimer’s disease [8,9,10]. Betzen et al. reported that oxidative stress induced by superoxide, peroxynitrite or hydrogen peroxide stimulated the NMDAR in bEnd3 cells and then decreased the monolayer impedance [13]. Furthermore, oxidative stress is known to have a co-relationship with neurodegenerative disorders such as Alzheimer’s disease, Parkinson’s disease and amyotrophic lateral sclerosis since the brain is more vulnerable to oxidative stress compared to other organs, and thus, is more susceptible to damage by these means [14]. Chiang et al. reported that the activation of NMDA receptors plays a critical role in learning and memory [15]. Additionally, they found that NMDA receptor enhancers such as sodium benzoate changed the activity of antioxidants, and thus, affected to the physiological status of the brain [15]. The aggravation of neurodegenerative disorders has a diverse relationship with oxidative stress in the brain [15,16,17,18]. Oxidative stress in the brain leads to neuronal cell death in the brain; moreover, NMDAR expression, mediated by oxidative stress, is believed to be a driving force of synapse dysfunction [22]. Our study also showed that oxidative stress increased the expression of the NMDAR1 protein in both SH-SY5Y and U87MG cells according to the increase in intracellular ROS levels, as shown in Figure 7, Figure 8, Figure 9 and Figure 10 and Figure S3. As shown in Figure 4, bCDsuMema nanoparticles exhibited ROS-sensitivity, i.e., the memantine release rate was increased according to the increase in ROS in the release media. In addition, the bCDsuMema nanoparticles suitably suppressed the expression levels of the NMDAR1 protein in both SH-SY5Y and U87MG cells (Figure 7 and Figure S3). Kamat et al. reported that the inhibition of oxidative stress and/or synapse dysfunction induced in the NMDAR can be applicable in the treatment of Alzheimer’s disease [22]. Hu et al. reported that antioxidants such as Kukoamine A efficiently suppress the expression of NMDAR in SH-SY5Y cells and modulate the apoptosis-related proteins [51]. Furthermore, the pretreatment of natural caffeoylquinic acid derivatives against SH-SY5Y cells attenuates hydrogen peroxide-induced apoptosis and oxidative stress [52]. Rosini et al. also reported that ferulic acid-memantine conjugates represent the suppression of NMDAR-mediated neurotoxic events that are mediated by amyloid-β burden and oxidative stress [53]. bCDsuMema nanoparticles suitably suppressed the ROS-derived expression of NMDAR1 protein in cells, as shown in Figure 7 and Figure 10. Our study showed that bCDsuMema nanoparticles have ROS-sensitivity, delivery capacity across the BBB and anti-Alzheimer’s disease activity.

## 4. Materials and Methods

### 4.1. Chemicals

Memantine HCl, succinyl β-cyclodextrin (bCDsu), N-(3-dimethylaminopropyl)-N’-ethylcarbodiimide hydrochloride (EDAC), N-hydroxy succinimide (NHS), 3-(4,5-dimethyl-2-thiazolyl)-2,5-diphenyl-2H-tetrazolium bromide (MTT) and hydrogen peroxide (H_2_O_2_) were purchased from Sigma Aldrich Chem. Co. (St. Louis, MO, USA). Thioketal diamine and thioketal dicarboxylic acid were purchased from RuixiBiotech Co. Ltd. (Xi’an, China). Chlorin e6 (Ce6) was purchased from Frontier Sci. Co. (Logan, UT, USA). Dialysis membranes (Molecular weight cutoffs size (MWCO): 1000, 2000 and 8000 g/mol) were purchased from Spectra/Pro^TM^ Membranes. A syringe filter (0.8 µm, Millex^®^ AA, MF-Millipore^TM^ MCE Membrane) was purchased from Merck Millipore Ltd. (Carrigtwohill, IRL). Dimethyl sulfoxide (DMSO), triethyl amine (TEA) and other organic solvent were used at an ultra-pure grade.

### 4.2. Synthesis of bCDsu-Thioketal-Memantine Conjugates

bCDsu-thioketal amine conjugates: 183 mg of succinyl β-cyclodextrin (bCDsu) was dissolved into 10 mL of H_2_O/DMSO mixtures (1/9). Seven equivalent quantities of EDAC and NHS were added to this solution and then stirred magnetically for 9 h. Following this, 680 mg of thioketal diamine (35 equivalents mole vs. bCDsu, 5 equivalents vs. each carboxylic acid of bCDsu) was dissolved in 10 mL DMSO and then added to bCDsu solution. This solution was magnetically stirred for 24 h. To obtain the synthesized conjugates, reactants were introduced into a dialysis tube (MWCO = 2000 g/mol) and then dialyzed against water for 2 days. To remove the organic solvent, deionized water was exchanged at 2–3 h intervals for 2 days. The resulting solution was freeze-dried for 2 days and then lyophilized power was obtained as a bCDsu-thioketal amine conjugate, as shown in Figure 1.

bCDsu-thioketal-memantine conjugates: for the synthesis of bCDsu-thioketal-memantine conjugates, memantine-thioketal carboxylic acid conjugates were primarily synthesized. First, 22.4 mg of thioketal dicarboxylic acid was dissolved in 10 mL DMSO with 1 equivalent quantity of EDAC and NHS with trace amounts of TEA. To this solution, 1 equivalent quantity of memantine HCl (21.6 mg) was added and then stirred for 12 h to obtain memantine-thioketal carboxylic acid. To activate the carboxylic acid end of the memantine-thioketal carboxylic acid, 19.2 mg of EDAC and 11.5 mg of NHS was added to the solution. This solution was further stirred for 6 h at room temperature. Following this, 153 mg of bCDsu-thioketal amine, dissolved in 10 mL of DMSO, was added to this solution and then magnetically stirred for 24 h. Finally, the resulting solution was introduced into a dialysis tube (MWCO: 2000 g/mol) and then dialyzed against deionized water for 2 days with an exchange of water at 2–3 h intervals. bCDsu-thioketal-memantine conjugates (abbreviated as bCDsuMema conjugates) were obtained by lyophilization for 2 days. The yield of final product was approximately 92 wt.%. Yield = ((weight of bCDsu-thioketal-memantine conjugates)/(weight of memantine-thioketal carboxylic acid conjugates + weight of bCDsu-thioketal amine conjugates)) × 100.

### 4.3. H Nuclear Magnetic Resonance (NMR) Spectra Measurement

Chemical composition and synthesis procedures were monitored using ^1^H NMR spectra (500 mHz superconducting Fourier transform (FT)-NMR spectrometer, Varian Unity Inova 500 MHz NB High-Resolution FT NMR; Varian Inc., Santa Clara, CA, USA). Each of the chemicals in the synthesis procedures were dissolved in DMSO or D_2_O/DMSO mixtures and then analyzed.

### 4.4. Fabrication of bCDsuMema Nanoparticles

Quantities of 20 mg of bCDsuMema conjugates (20 mg) were dissolved in 5 mL DMSO/water mixtures (4/1 *v*/*v*). This solution was introduced into the dialysis tube (MWCO = 2000 g/mol) and then dialyzed against deionized water. To prevent saturation of the solvent, deionized water was exchanged at 3 h intervals for 12 h and then at 6 h intervals for 24 h. Following this, the volume of dialyzed solution was adjusted to 20 mL (1 mg nanoparticles/mL water) and used for analysis or drug release experiments.

The drug contents in the nanoparticles were measured as follows: 5 mg nanoparticle solution prepared as described above was reconstituted in 5 mL of phosphate-buffered saline (PBS, 0.01 mM pH 7.4) and then added H_2_O_2_ (the final concentration of H_2_O_2_ was 20 mM). This solution was incubated more than 48 h. Following this, the absorbance of the resulting solution was measured with a UV spectrophotometer (UV-1601PC UV/VIS spectrophotometer, Shimadzu CO., Kyoto, Japan) at 230 nm (Figure S4). For comparison, the free memantine and bCDsu-thioketal amine conjugates, dissolved in phosphate-buffered saline (PBS, 0.01 M, pH 7.4) with H_2_O_2_, were also measured.

Memantine content (wt.%) = (Memantine weight/total weight of nanoparticle)/100.(1)

### 4.5. Characterization of Nanoparticles

The morphologies of nanoparticles were observed using transmission electron microscopy (TEM) (H-7600, Hitachi Instruments Ltd., Tokyo, Japan). One drop of the nanoparticle solution was placed onto the carbon-film-coated grid. Then, this was dried at room temperature. The observation was carried out at 80 kV.

Particle sizes were measured with a Zetasizer Nano-ZS (Malvern, Worcestershire, UK). For measurement of the particle sizes, the concentrations of nanoparticles were adjusted to 0.1–1 mg/mL.

### 4.6. Drug Release Study

Nanoparticles (5 mg) prepared as described above were reconstituted into 5 mL PBS (0.01 M, pH 7.4) in the absence or presence of H_2_O_2_. This solution was introduced into the dialysis tube (MWCO = 2000 g/mol) and then put into a conical tube with 45 mL PBS (final H_2_O_2_ concentration was adjusted to 0.1 mM~10 mM). This solution was incubated in a shaker incubator (SI-600R, Jeiotech Co., Daejeon, Korea) at 100 rpm and 37 °C. The whole media was taken and replaced with fresh media to prevent saturation of the drug. Media were used to measure the memantine concentration using a UV spectrophotometer (UV-1601PC UV/VIS spectrophotometer, Shimadzu CO., Kyoto, Japan) at 230 nm. To avoid interference by conjugates, bCDsu-thioketal amine conjugates were also adapted for the release study, and their release media were used for the blank test. All the results were triplicated and expressed as mean ± standard deviation (S.D.).

### 4.7. Cell Culture

SH-SY5Y neuroblastoma cells were purchased from American Type Culture Collection (ATCC, Manassas, VA, USA), maintained in Eagle’s minimum essential medium/F12 (1/1 mixtures (Gibco, Grand Island, NY, USA)), and supplemented with 10% heat-inactivated fetal bovine serum and 1% penicillin/streptomycin. U87MG glioblastoma cells were purchased from the Korean Cell Line Bank (Seoul, Korea). U87MG cells were maintained with Dulbecco’s minimum essential medium (DMEM, Gibco, Grand Island, NY, USA) supplemented with 10% fetal bovine serum and 1% antibiotics. All cells were cultured in a 5% CO_2_ incubator at 37 °C.

### 4.8. MTT Assay

Cells (1 × 10^4^ cells/well) seeded in 96 wells were cultured overnight in a CO_2_ incubator (5% CO_2_) at 37 °C. After that, the cells were exposed to H_2_O_2_ (final concentration: 100 µM) in serum-free media for 6 h or 24 h. To assess the cytotoxicity of the nanoparticles, cells (1 × 10^4^ cells/well) were treated with 5 and 10 µg/mL of memantine or nanoparticles for 24 h. The viability of U87MG and SH-SY5Y cells was determined by EZ-CyTox (tetrazolium salt, WST-1) assay (Daeil Lab Inc, Seoul, Korea) (EZ3000). Absorbance was measured at 570 nm, and cell viability was expressed as the fraction of surviving cells relative to untreated controls. All cell culture experiments were triplicated and then expressed as average ± standard deviation (S.D.).

### 4.9. Antibodies

The primary antibodies used were anti-rabbit antibodies against NMDAR1 (ab109182), obtained from Abcam (Alomone Laboratories, Ltd., Jerusalem, Israel), and β–actin (a5316), obtained from Santa Cruz Biotechnology, Inc. (Dallas, TX, USA).

### 4.10. Western Blot Assay

For the analysis of NMDAR1 expression, cells were treated with H_2_O_2_ (final concentration: 100 µM) for 6 h or 24 h. To assess the effect of drugs on the expression of NMDAR1, cells were pre-treated with memantine, or nanoparticles were treated for 1 h and then exposed to H_2_O_2_ (final concentration: 100 µM) for 6 h. After that, cells were harvested, washed twice with ice-cold PBS (0.01 M, pH 7.4), resuspended in lysis buffer and sonicated briefly. The protein extraction buffer consisted of 50 mM Tris-HCl (pH 7.2), 5 mM EDTA, 150 mM NaCl, 1% Nonidet P-40, 0.1% SDS, protease inhibitor cocktail (GenDEPOT, P3100-001) and phosphatase inhibitor cocktail (GenDEPOT, P3200-001). After centrifugation, supernatants were obtained containing the protein extracts; the protein concentrations were measured using a Pierce^®^ BCA Protein Assay Kit (Pierce Biotechnology, Inc., Rockford, IL, USA). Concentrations of 30 μg of protein were separated on 12% sodium dodecyl sulfate polyacrylamide gels, and the proteins were transferred onto nitrocellulose membranes. The blots were blocked at room temperature for 2 h with 5% skim milk in PBS buffer containing 0.1% Tween-20 (PBST). The blot was then incubated with the primary antibody (1:2000) (antiNMDAR1 antibody, abcam co., Cambridge, MA, USA) overnight at 4 °C, followed by incubation of the secondary antibody (1:2500), followed by incubation with anti-rabbit horseradish peroxidase-conjugated antibodies, as described previously [54]. The labeling was visualized using an enhanced chemiluminescence system.

### 4.11. Fluorescence Microscopy

Intracellular ROS levels were measured in accordance with the manufacturer’s instructions. U87MG cells (1 × 10^5^) or SH-SY5Y cells (1 × 10^5^) cells were seeded in 4-well chambers and, following 24 h incubation at 37 °C, the cells were treated with 100 μM H_2_O_2_ for 6 h. After that, the cells were washed twice with PBS and were then stained with CM-H_2_DCFDA. These were washed twice with PBS and then fixed with 4% paraformaldehyde for 10 min at room temperature. These were washed twice with PBS and then immobilized with ProLong Gold Antifade Reagent with DAPI.

For observation of the NMDAR1 changes in cells, U87MG cells (1 × 10^5^) or SH-SY5Y cells (1 × 10^5^) were seeded in 4-well chambers. Then, the cells were pre-treated with memantine or bCDsuMema nanoparticles for 1 h and then treated with 100 μM H_2_O_2_ for 6 h. Following this, the cells were washed with PBS twice and then fixed with 4% paraformaldehyde for 10 min at room temperature. Following this, the cells were washed with PBS twice and were then stained with anti-NMDAR1 antibody diluted 1:300 in blocking buffer for 24 h at 4 °C. After washing, the cells were incubated with a Cy3 (red fluorescence) goat anti-rabbit antibody (Life Technologies, Carlsbad, CA, USA), diluted 1:500 in blocking buffer for 2 h, and a Phalloidin was added for incubation (F-actin, Alexa Fluor 488, green fluorescence) for 30 min. After washing, the coverslips were mounted onto microslides using a ProLong Gold Antifade Reagent with DAPI (Life Technologies Corporation). Images were captured using an LSM 510 confocal microscope (Carl Zeiss, Jena, Germany).

### 4.12. In Vivo Fluorescence Imaging

For fluorescence imaging of the nanoparticles, nude BALb/C mice (male, 20 g, 5 weeks old) was used. Quantities of 20 mg of bCDsuTHCe6 or bCDsuCe6 nanoparticles were reconstituted in 4 mL PBS (0.01 M, pH 7.4). This solution was sterilized through filtration with a 0.8 µm syringe filter and then administered intravenously via the tail veins of the mice. The injection volume was 100 µL. After 24 h of injection, the mice were sacrificed and each organ was observed with a Maestro^TM^ 2 small animal imaging instrument.

### 4.13. Statistical Analysis

Statistics of the experimental results were evaluated with the Student’s *t*-test and *p* values lower than 0.05 were considered as statistically significant.

## 5. Conclusions

Nanoparticles of bCDsuMema conjugates were synthesized to investigate their effect on the expression of the NMDAR1 protein in SH-SY5Y neuroblastoma cells and U87MG glioblastoma cells. bCDsuMema nanoparticles had small particle sizes less than 100 nm and their morphology was spherical. H_2_O_2_, a typical ROS, disintegrated or swelled the nanoparticles in the aqueous solution, and then release rate of memantine was accelerated according to the concentration of H_2_O_2_. In the in vivo animal imaging study, thioketal-decorated nanoparticles showed efficient brain targeting. Memantine or bCDsuMema nanoparticles had practically no effect on the viability of the cells, while the cells were affected by treatment with H_2_O_2_. The western blot assay showed that the oxidative stress produced in cells using H_2_O_2_ increased the expression of the NMDAR1 protein in both SH-SY5Y and U87MG cells. Memantine and bCDsuMema nanoparticles both efficiently suppressed the ROS-mediated expression of the NMDAR1 protein in cells. In the fluorescence microscopy observations, H_2_O_2_ treatment increased the intracellular ROS levels, and then the oxidative stress increased NMDAR1 protein expression in brain cells. When cells were treated with memantine or bCDsuMema nanoparticles, the oxidative-mediated expression of the NMDAR1 protein in cells was significantly decreased, indicating that bCDsuMema nanoparticles have the capacity to suppress NMDAR1 expression in brain cells, which has relevance to applications in Alzheimer’s disease. These results suggested that bCDsuMema nanoparticles are a promising candidate for the inhibition of Alzheimer’s disease.

## Figures and Tables

**Table 1:** Characterization of nanoparticles of bCDsuMema conjugates.

	Memantine Contents (%, *w/w*)		Particle Size (nm)
	Theoretical *	Experimental	
bCDsuMema conjugates	9.4	9.1	82.8 ± 12.3

* Theoretical contents of memantine in the bCDsuMema conjugates were calculated based on the feeding amount of memantine in the synthesis scheme.

**Table 2:** Changes of particle size distribution caused by the addition of H_2_O_2_ (derived from Figure 4a).

H_2_O_2_ Contents (mM)	Particle Size Distribution		Polydispersity
	Diameter ± S.D. (nm)	% Intensity	
1	103.7 ± 35.68	93.1	0.271
	4603 ± 826.2	6.9	
5	31.32 ± 7.833	9.7	0.283
	155.3 ± 74.28	90.3	
10	137.5 ± 80.97	96.9	0.268
	4599 ± 828.3	3.1	

## Data Availability

The presented in this study are available within the manuscript and the Supplementary Materials.

## Supplementary Materials

The following are available online at https://www.mdpi.com/article/10.3390/ijms222212309/s1.
