# Supplementary material for: The Effect of Oxidative Stress and Memantine-Incorporated Reactive Oxygen Species-Sensitive Nanoparticles on the Expression of N-Methyl-d-aspartate Receptor Subunit 1 in Brain Cancer Cells for Alzheimer’s Disease Application
Source: Int J Mol Sci. 2021 Nov 15;22(22):12309. doi: 10.3390/ijms222212309 (PMC8619842; doi:10.3390/ijms222212309)
Supplement: Supplementary file 1 [file ijms-22-12309-s001.zip › ijms-1425685-supplementary.pdf]

Supporting Materials

Materials and methods

Synthesis of bCDsu-thioketal-Ce6 conjugates

bCDsu-thioketal-Ce6 conjugates: bCDsu-thioketal amine conjugates was prepared as described in 4.2 Synthesis of bCDsu-thioketal-memantine conjugates. 30 mg of Ce6 dissolved in 5 ml DMSO was mixed with 9.6 mg EDAC and 5.8 mg NHS. This solution was magnetically stirred for 6 h. Following this, 153 mg bCDsu-thioketal amine dissolved in 10 ml DMSO was added to this solution and then magnetically stirred for 24 h. Finally, resulting solution was introduced into dialysis tube (MWCO: 2000 g/mol) and then dialyzed against deionized water for 2 days with exchange of water at 2-3 h intervals. bCDsu-thioketal-Ce6 conjugates (bCDsuTHCe6 conjugates) was obtained by lyophilization for 2 days. Final product was abbreviated as bCDsuTHCe6. Yield of final product was higher than 93 wt.%. Yield = [(weight of bCDsu-thioketal-Ce6 conjugates)/(weight of Ce6 + weight of bCDsu-thioketal amine conjugates)] $\times$100.

bCDsu-Ce6 conjugates: Prior to synthesis of bCDsu-Ce6 conjugates, Ce6-thioketal amine conjugates were synthesized. Briefly, 60 mg Ce6 dissolved in 5 ml DMSO was mixed with 19.2 mg EDAC and 11.5 mg NHS. This solution was magnetically stirred for 6 h. Following this, thioketal amine (19.4 mg) was added to this solution and then stirred magnetically for 24 h. Following this, this solution was dropped into 20 ml deionized water and then introduced into dialysis tube (MWCO: 500 g/mol). This was dialyzed against deionized water for 1 day. Resulting solution was lyophilized for 2 days. Final product was abbreviated as bCDsuCe6. Yield of final product was higher than 94 wt.% (w/w). Yield = [(weight of Ce6-thioketal amine conjugates)/(weight of Ce6 + weight of thioketal diamine)] $\times$100.

For synthesis of bCDsu-Ce6 conjugates, 92 mg bCDsu was dissolved into 5 ml of H_2O/DMSO mixtures (1/9). 1 equivalents EDAC and NHS were added to this solution and then stirred magnetically for 9 h. Following this, 39 mg of Ce6-thioketal amine conjugates dissolved in 5 ml DMSO was added to this solution and then stirred magnetically for 24 h. Finally, resulting solution was introduced into dialysis tube (MWCO: 2000 g/mol) and then dialyzed against deionized water for 2 days with exchange of water at 2-3 h intervals. Final product was abbreviated as bCDsu-Ce6 conjugates. Yield of final product was higher than 92 wt.% (w/w). Yield = [(weight of bCDsu-Ce6 conjugates)/(weight of Ce6-thioketal amine conjugates + weight of bCDsu)] $\times$100.

UV spectrophotometer measurement of memantine

Memantine HCl dissolved in DMSO was diluted with PBS (0.01 M, pH 7.4) more than 10 times. This was used to measure absorbance scan with UV spectrophotometer (UV-1601PC UV/VIS spectrophotometer, Shimadzu CO., Kyoto, Japan) at 200 ~ 700 nm (Figure s4).

Preparation of nanoparticles

bCDsuTHCe6 or bCDsuCe6 conjugates (20 mg) were dissolved in 5 mL DMSO/water mixtures (4/1 *v*/*v*) and then prepared nanoparticles as described in Materials and methods. Briefly, organic solvents were introduced into the dialysis tube (MWCO = 2000 g/mol) and then dialyzed against deionized water. To prevent saturation of solvent, deionized water was exchanged every 3 h intervals for 12 h and then 6 h intervals for 24 h. Following this, the volume of dialyzed solution was adjusted to 20 mL (1 mg nanoparticles/mL water) used for analysis or drug release experiment.

The Ce6 contents in the nanoparticles were measured as follows: 5 mg nanoparticle solution prepared as described above was reconstituted in 5 ml of phosphate-buffered saline (PBS, 0.01 mM pH 7.4) and then added H_2O_2 (The final concentration of H_2O_2 was 20 mM). This solution was incubated more than 48 h and then diluted with DMSO ten times. Following this, resulting solution was measured absorbance with UV spectrophotometer (UV-1601PC UV/VIS spectrophotometer, Shimadzu CO., Kyoto, Japan) at 664 nm. For comparison, free memantine and bCDsu-thioketal amine conjugates or bCDsu dissolved in phosphate-buffered saline (PBS, 0.01 M, pH 7.4) with H_2O_2 were also measured.

Ce6 content (wt.%) = (Ce6 weight/total weight of nanoparticle)/100.

Western blot assay

For analysis of NMDAR1 expression, cells were treated with H_2O_2 (Final concentration: 100 μM) for 6 h or 24 h. To assess the effect of drug on the expression of NMDAR1,cells were pre-treated with memantine or nanoparticles were treated for 1 h and then exposed to H_2O_2 (Final concentration: 100 μM) for 6 h. After that, cells were harvested, washed twice with ice-cold PBS (0.01 M, pH 7.4), resuspended in lysis buffer, and sonicated briefly. Protein extraction buffer consists of 50 mM Tris-HCl (pH. 7.2), 5 mM EDTA, 150mM NaCl, 1% Nonidet P-40, 0.1% SDS, protease inhibitor cocktail (GenDEPOT, P3100-001) and phosphatase inhibitor cocktail (GenDEPOT, P3200-001). After centrifugation, supernatants were obtained containing the protein extracts; the protein concentrations were measured using

a Pierce® BCA Protein Assay Kit (Pierce Biotechnology, Inc., Rockford, IL, USA). 30 μg concentrations of protein were separated on 12% sodium dodecyl sulfate polyacrylamide gels, and the proteins were transferred onto nitrocellulose membranes. The blots were blocked at room temperature for 2 h with 5% skim milk in PBS buffer containing 0.1 % Tween-20 (PBST). The blot was then incubated with the primary antibody (1:2000) (antiNMDAR1 antibody, abcam co., Cambridge, MA, USA) overnight at 4 ℃, followed by incubation of the secondary antibody (1:2500), followed by incubation with anti-rabbit horseradish peroxidase-conjugated antibodies. The labeling was visualized using an enhanced chemiluminescence system.

Results

As shown in Figure S1, 1H NMR spectra showed specific peaks of bCDsu between 2 ~ 5 ppm. This can be used to compared with conjugates. Specific peaks of memantine HCl was observed at 0.8 ppm of methyl protons between 0.6 ~ 3.6 ppm. Thioketal diamine was also observed at 1.6 ppm of methyl proton and 2.8~3.0 ppm of ethyl protons. Thioketal dicarboxylic acid was observed at 1.6 ppm of methyl protons.

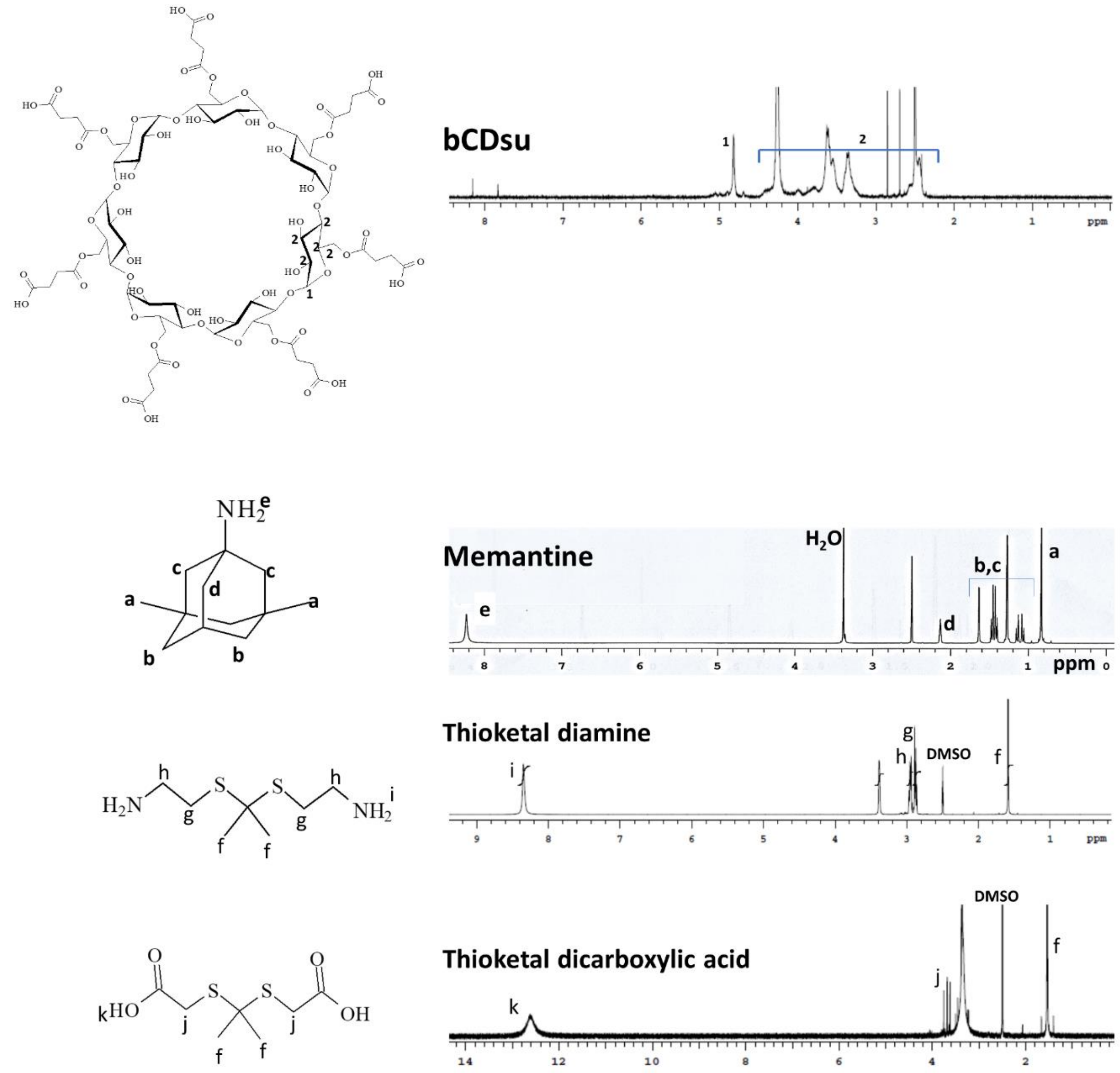

Figure S1. Chemical structures and ^{1}H NMR spectra of bCDsu, memantine, thioketal diamine and thioketal dicarboxylic acid.

To observe animal imaging, chlorin e6 (Ce6), a fluorescent dye, was conjugated with bCDsu-thioketal amine instead of memantine-thioketal carboxylic acid as shown in Figure S2(a). As shown in Figure S2(a), specific peaks of bCDsu was confirmed at 2 ~ 4.6 ppm and 4.8 ppm. Specific peaks of thioketal amine were confirmed at 1.6 ppm of methyl group and 2 ~ 3 ppm of ethyl group. Furthermore, specific peaks of Ce6 was confirmed between 1 and 10 ppm, i.e. peaks of 1.2 ppm and 9~10 ppm was derived from Ce6. Furthermore, bCDsuCe6 was also synthesized to compare bCDsuTHCe6 as shown in Figure S2(b). To attach Ce6 with bCDsu, Ce6 was conjugated with thioketal diamine and then Ce6-thioketal amine was conjugated again with bCDsu as shown in Figure S2(b). As shown in Figure S2(b), specific peaks of bCDsu was confirmed at 2 ~ 4.6 ppm and 4.8 ppm. Specific peaks of thioketal amine were confirmed at 1.6 ppm of methyl group. Furthermore, peaks of Ce6 were confirmed at 1.0 ~ 1.2 ppm, 6.0 ~6.2 ppm and 9~10 ppm, respectively. Ce6 contents of bCDsuTHCe6 or bCDsuCe6 were 15.6 % (w/w) and 21.8 % (w/w), respectively. Nanoparticles of bCDsuTHCe6 or bCDsuCe6 were prepared by dialysis method. Particle sizes of bCDsuTHCe6 or bCDsuCe6 were 106.8±10.8 nm and 89.6±8.2 nm, respectively.

Figure S3 shows western blot analysis of NMDAR1 protein expression in U87MG cells and SH-SY5Y cells. These results are raw data of Figure 7.

Figure S4 shows the UV spectra of memantine in PBS. As shown in Figure S4, memantine has maximum peak at 230 nm.

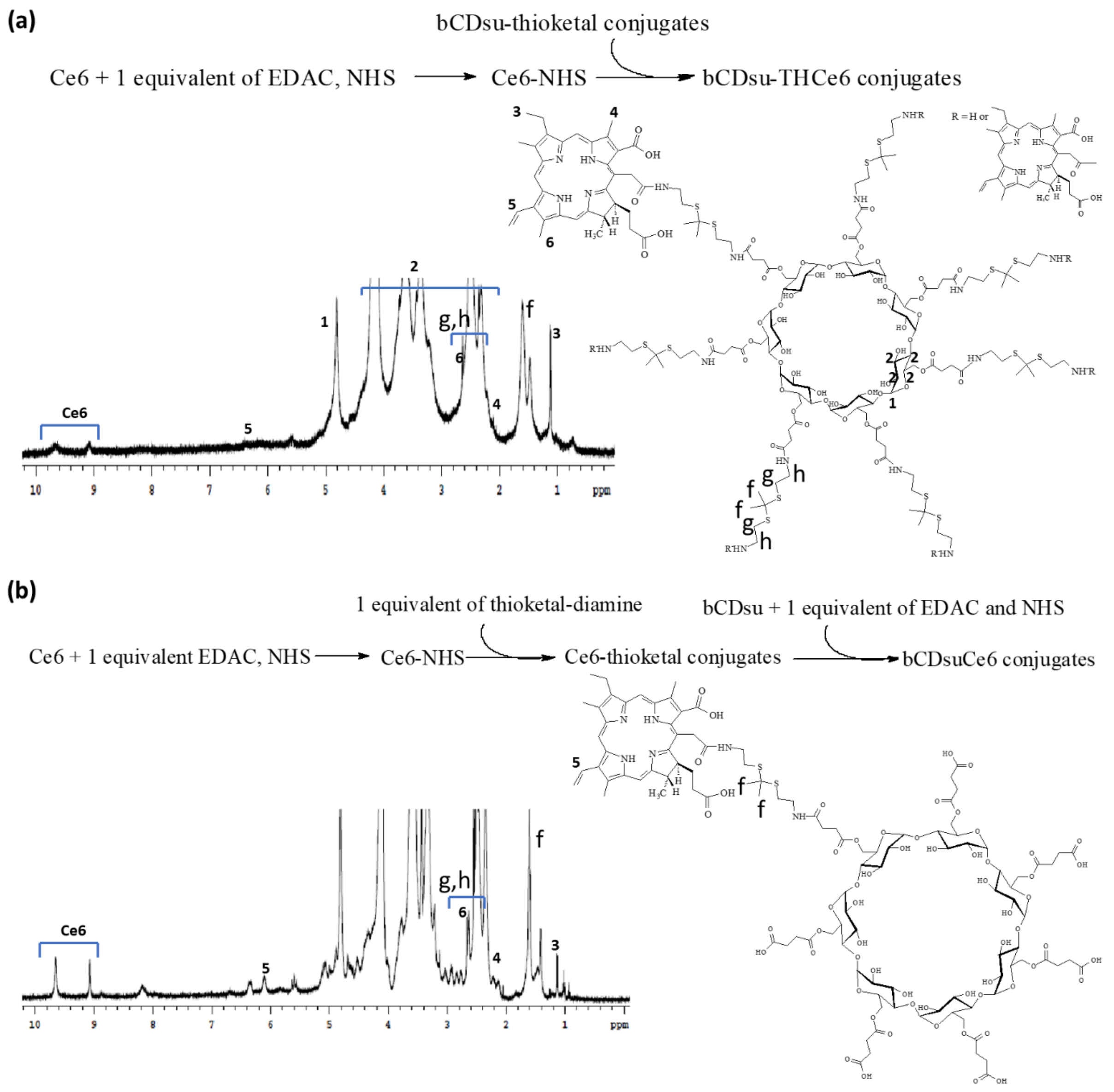

Figure S2. Synthesis scheme and ^{1}H NMR spectra of bCDsuTHCe6 conjugates (a) and bCDsuCe6 conjugates (b).

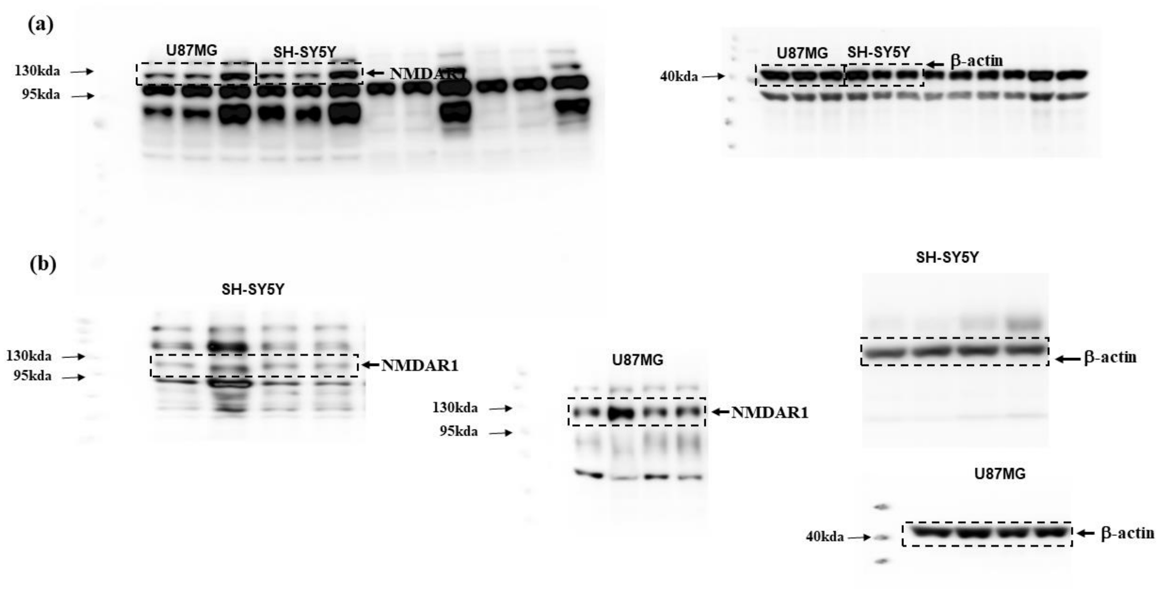

Figure S3. The effect of H_2O_2 and memantine on the NMDAR1 expression of cells. (a) The effect of H_2O_2 on the NMDAR1 protein expression in U87MG and SH-SY5Y cells. (b) The effect of memantine on the H_2O_2-induced NMDAR1 protein expression in U87MG and SH-SY5Y cells. These are raw data of Figure 7.

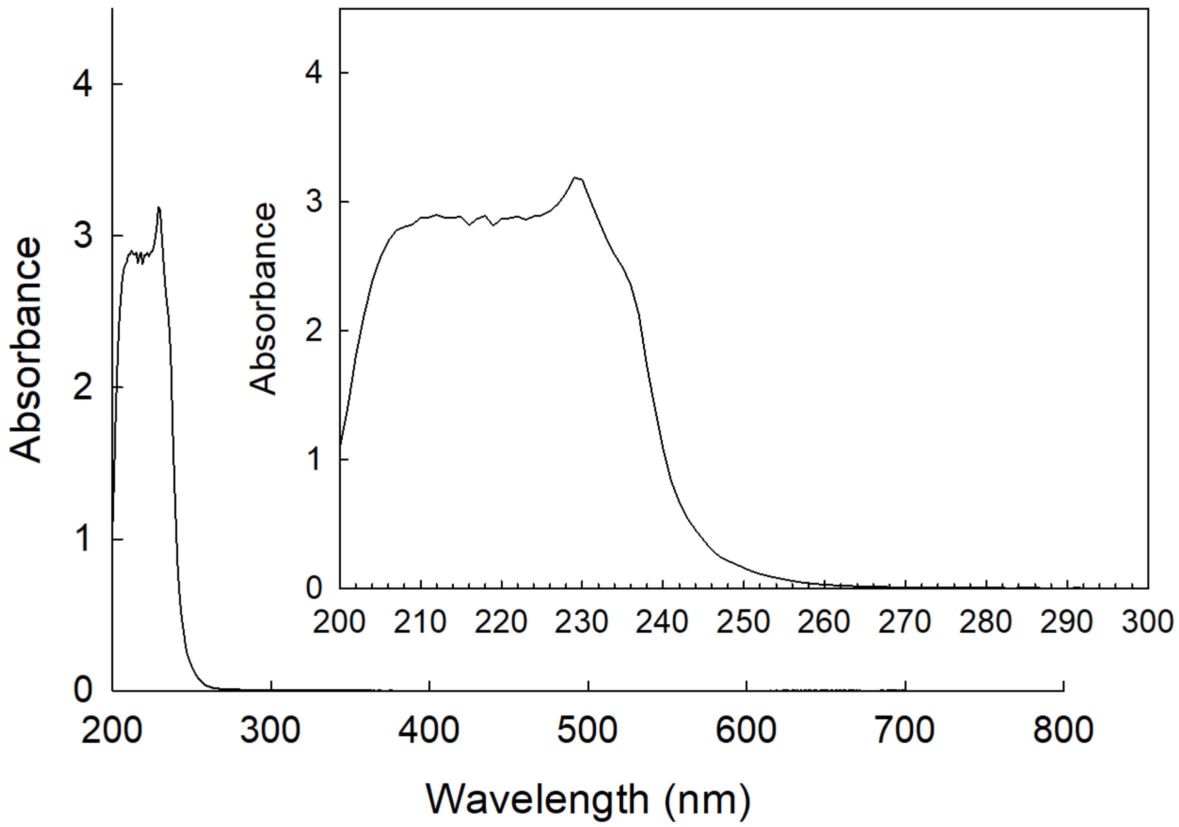

Figure S4. UV spectra of memantine (0.5 mg/ml) in PBS (pH 7.4, 0.01 M).
